# Transition in availability of improved sanitation facilities and its effect on diarrhoeal disease in India: evidence from longitudinal data

**DOI:** 10.1093/inthealth/ihac082

**Published:** 2023-01-10

**Authors:** N Brahmanandam, Milind Sadashiv Bharambe

**Affiliations:** Doctoral student, Development Studies, Department of Development Studies, International Institute for Population Sciences, Deonar, Govandi Station Road, Mumbai 400088, Maharashtra, India; Doctoral student, School of Development Studies, Tata Institute of Social Sciences, Deonar, Mumbai 400088, Maharashtra, India

**Keywords:** diarrhoea, India, longitudinal data, morbidity, sanitation, socio-economic status

## Abstract

**Background:**

Poor sanitation, such as open defecation, is a major public health concern in India, causing diarrhoea and other infectious diseases. So far, few studies have linked poor sanitation with diarrhoea using longitudinal data. In this context, this study assesses the transition in availability of household sanitation facilities and its effect on diarrhoeal morbidity.

**Methods:**

We used two waves of longitudinal data from the India Human Development Survey, conducted in 2004–2005 and 2011–2012, and based on 34 131 followed-up households using a two-stage stratified random sampling method. In the first stage, multinomial logistic regression was used to assess socio-economic factors contributing to the transition in the availability of household sanitation facilities. In the second stage, multivariate linear regression was performed to examine the effect of the change in the availability of household sanitation facilities on the prevalence of diarrhoeal morbidity. All the analysis in this study was carried out by using Stata version 13 software.

**Results:**

The findings reveal that the practice of open defecation was continued to be higher among lower socio-economic households than better-off socio-economic households in both 2004–2005 and 2011–2012. The proportion of household members who fell sick due to diarrhoea morbidity has decreased significantly (β=−0.06, p<0.04) among households that switched from open defecation in 2004–2005 to improved sanitation facilities in 2011–12, compared to households that continued to practice open defecation in both periods (2004–2005 and 2011–2012). The share of household members who fell sick due to diarrhoeal morbidity was significantly lower (β=−0.09, p<0.001) among the households who adopted improved toilet facilities in both periods (2004–2005 and 2011–2012) as compared with the households who continued to defecate openly in both periods, net of other covariates.

**Conclusions:**

Our findings show that there is a need to strengthen existing policies focusing on lower socio-economic groups to improve sanitation and eliminate its related diseases. In particular, the ongoing ‘Clean India Mission’ should play a critical role in promoting sanitation for all.

## Introduction

As of 2016, diarrhoea was the eighth leading cause of death globally, accounting for >1.6 million deaths, and is accountable for more than one-quarter of all deaths in children <5 y of age (26.93%).^1^ The practice of open defecation leads to the transmission of pathogens that cause diarrheal diseases. The failure to effectively manage human excreta causes diarrhoea and a wide range of health problems and increases the burden of several diseases.^2–4^ Systematic reviews, meta-analysis and cross-sectional studies have shown that sanitation interventions can be effective in reducing a range of important health outcomes, including diarrhoeal diseases, soil-transmitted helminth infections, child mortality and child malnutrition, including underweight and stunting.^5–9^

Women are more vulnerable to open defecation health concerns such as hookworm infection, resulting in maternal anaemia, which in turn is directly associated with adverse pregnancy outcomes.^10,11^ Open defecation has an adverse social impact on women and girls. The lack of household toilet facilities forces many women and girls to travel long distances from their home to find private open places to defecate and manage their menstrual needs, which makes them more vulnerable to health issues, abuse and various forms of violence.^12^ The heightened fear, shame and helplessness are common in girls and women in lower- and lower-middle-income countries where open defecation continues to persist.^13^

The main aim of Sustainable Development Goal 6 (SDG-6) is to provide equitable access to water and sanitation for all by 2030.^8^ However, the problem of open defecation remains prevalent in developing countries and in India.^14^ Since 2000, the global rate of open defecation has declined from 21% to 9%.^14^ Despite a decline in open defecation, as of 2016, 1 billion people practiced open defecation and >600 million did not have access to even a basic level of drinking water.^8^ In India, the prevalence of open defecation decreased from 52% to 30% between 2015–2016 and 2019–2021. However, it is considerably higher in rural areas (35%).^15^ Thus, despite considerable improvement in sanitation facilities, poor sanitation still persists in India.^15^

Studies have reported that the higher prevalence of open defecation in India is a puzzle that is not explained by development indicators.^16^ For instance, neighbouring Bangladesh's per capita income is only half that of India and it has a higher percentage of the population living below the poverty line than India.^16^ Even though Bangladesh performs lower than India on economic indicators, its prevalence of open defecation in rural areas is only 4%, which is much lower than India's 35% in rural areas.^15,16^ Thus the explanation for the sanitation behaviour of households in India goes beyond the development indicators. Most often, household sanitation behaviour is associated with ritual purity, pollution and caste among Hindu households.^17–20^ In rural India, Hindu households are reluctant to empty the pit latrine, because it is associated with the work of lower castes.^20^ The social, cultural and ritual practices associated with Hindu households lead them to defecate in open places.

All the previous studies have assessed the socio-economic and cultural determinants of sanitation behaviour with cross-sectional data.^16,19,20^ Before the current study, only one study using longitudinal data reported socio-economic determinants of switching to sanitation facilities.^17^ Compared with previous cross-sectional and panel data studies, our study makes a significant contribution. We examine the transition in the availability of sanitation facilities by socio-economic determinants using the large-scale longitudinal data of the India Human Development Survey (IHDS). In addition, the study also assesses the effect of transition in household adoption of improved sanitation facilities on diarrhoea morbidity at the household level, net other socio-economic covariates.

## Methodology

### Data

The present study used two waves of a longitudinal data from the IHDS (2004–2005 and 2011–2012). The IHDS is a nationally representative survey carried out in collaboration with researchers at the University of Maryland and the National Council of Applied Economic Research (NCAER), New Delhi, India. The first wave of the survey (IHDS-I) was administered to a nationally representative sample of 41 554 households. In the second wave, 83% of the households from IHDS-I were reinterviewed.^21^ These households were spread across 33 states and union territories: 384 districts, 1503 villages and 971 urban blocks located in 276 towns and cities. Villages in rural areas and census enumeration blocks in urban areas (comprising 150–200 households) formed the primary sampling unit (PSU) from which the households were selected. Stratified random sampling was used to select the rural sample in 1503 villages. To draw a random sample of urban households, all urban areas in a state were listed in the order of their size, with the number of blocks drawn from each urban area allocated based on probability proportional to size. A detailed description of the sampling design and data collection method is discussed elsewhere.^21,22^

For the analytical purpose of this study, the total sample size of reinterviewed households from IHDS-I was 34 131. Both surveys collected household information on income, education, health, consumption expenditures, fertility and family planning. These surveys also collected information on adoption of improved sanitation facilities and diarrhoea morbidity at the household level. The questions were asked to all members of households regarding diarrhoea morbidity during the month preceding the survey. Both surveys covered all the states and union territories of India except the Andaman and Nicobar Islands and Lakshadweep Islands.

### Variables

There are two outcome variables in this study. The first outcome variable is a multinomial variable comprising four categories indicating the transition nature of households that adopted improved sanitation facilities: remained in open defecation status in 2004–2005 and 2011–2012, openly defecated in 2004–2005 but adopted improved toilet facilities in 2011–2012, adopted improved toilet facilities in 2004–2005 but defecated openly in 2011–2012 and adopted improved toilet facilities in 2004–2005 and 2011–2012. The second outcome variable is continuous: the percentage of household members who fell ill due to diarrhoea morbidity.

The variable of ‘transition in availability of improved sanitation facilities for a household’ is considered for two purposes. In the first stage, this multinomial variable was used as a dependent variable to assess the transition in improved sanitation facilities by households’ socio-economic status. Second, it was used as a predictor variable to determine the effect of transition to improved sanitation facilities on diarrhoeal morbidity at the household level. The socio-economic covariates considered for the models include place of residence (rural and urban), type of house (*kucha* and *pucca*), occupation of the head of the household (primary, secondary, tertiary and no occupation), education level of the head of the household (illiterate, primary, secondary and higher education), economic status of the household (poor and non-poor), social groups (general, other backward caste [OBC], scheduled caste [SC] and scheduled tribe [ST]), religious groups (Hindu, Muslim, Christian and Other) and regional categories (north, central, east, northeast, west and south).

### Statistical analysis

The analyses were carried out in two stages. In the first stage we used bivariate and χ^2^ tests to assess the change in improved sanitation facilities of households by different socio-economic characteristics. In the second stage, for the multinomial variable, i.e. transition in improved sanitation facilities of households, we applied the multinomial logistic regression and multiple classification analysis (MCA) conversion model to assess the association of different socio-economic variables to the transition to improved sanitation facilities of households.

### Multinomial logistic regression and MCA conversion model

In this study, multinomial logistic regression and the MCA conversion model are used to estimate the adjusted percentage of transition in improved sanitation facilities of households by their socio-economic characteristics.

The mathematical equation of MCA is as follows:


\begin{equation*}{Z}_{\mathrm{1}} = Log\left( {\frac{{{P}_{\mathrm{1}}}}{{{P}_{\mathrm{4}}}}} \right) = {a}_{\mathrm{1}} + \sum {b}_{{\mathrm{1}}j}*{X}_j
\end{equation*}

\begin{equation*}{Z}_2 = Log\left( {\frac{{{P}_{\mathrm{2}}}}{{{P}_{\mathrm{4}}}}} \right) = {a}_{\mathrm{2}} + \sum {b}_{{\mathrm{2}}j}*{X}_j\end{equation*}

\begin{equation*}{Z}_3 = Log\left( {\frac{{{P}_{\mathrm{3}}}}{{{P}_{\mathrm{4}}}}} \right) = {a}_{\mathrm{3}} + \sum {b}_{{\mathrm{3}}j}*{X}_j\end{equation*}

\begin{equation*}{P}_1\, + \,{P}_2\, + {P}_3\, + {P}_4\, = \,1\end{equation*}

where

ai i = 1, 2: constants;

bij i = 1, 2; j = 1, 2….n: multinomial regression coefficient;

*P*_1_=estimated probability of households continued to practice open defecation in 2005 and 2012;

*P*_2_=estimated probability of households openly defecated in 2005 but adopted improved toilet facilities in 2012;

*P*_3_=estimated probability of households adopted improved toilet facilities in 2005 but defecated openly in 2012; and

*P*_4_=estimated probability of households adopted improved toilet facilities in both periods, 2005 and 2012.

Here *P*_4_ is a reference category.

For the sake of simplicity in the interpretation of results, multinomial logistic regression coefficients are converted into adjusted percentages. The procedure consists of following steps:

Step 1:

By using regression coefficient and mean values of independent variables, the probability is computed as:

${P}_i = \frac{{\exp ({Z}_i)}}{{\{ 1 + \sum \exp ({Z}_i)\} }}$ , *i* = 1, 2, 3, 4 and *P*_4_=1−*P*_1_+*P*_2_+*P*_3_, where *Z* is the estimated value of response for all categories of each variable.

Step 2:

To obtain the percentage values, the probability *P* was multiplied by 100.^24^

In the second stage, for the continuous linear variable, i.e. the percentage of household members who fell ill due to diarrhoea morbidity, we used the multivariate linear regression to see the effect of transition to improved sanitation facilities of the households on diarrhoea morbidity. All the analyses for this article were carried out by using Stata version 13.1 (StataCorp, College Station, TX, USA).

## Results

### Transition in availability of improved sanitation facilities by socio-economic characteristics

Table 1 shows that less than half of households in India (46.2%) continued to practice open defecation from 2004–2005 to 2011–2012, while nearly one-third (32.2%) of the households espoused improved toilet facilities. Only 16% switched to improved toilet facilities in 2011–2012 from the practice of open defecation in 2004–2005, while 5% switched from improved toilet facilities in 2004–2005 to open defecation in 2011–2012.

**Table 1.** Transition in households’ improved sanitation facilities from 2004–2005 to 2011–2012 by different socio-economic characteristics in India

	Change in households’ sanitation				
Background characteristics	Openly defecated in 2005 and 2012	Openly defecated in 2005 and adopted improved toilet facilities in 2012	Adopted improved toilet facilities in 2005 and defecated openly in 2012	Adopted of improved toilet facilities in 2005 and 2012	n
Place of Residence					
Rural	57.0	16.1	5.3	21.6	23 642
Urban	13.7	16.5	4.8	65.0	10 489
Pearson χ^2^=6600, p=0.000					
Type of house					
*Kucha*	59.5	17.0	5.0	18.6	21 505
*Pucca*	18.9	14.7	5.4	61.0	12 626
Pearson χ^2^=7100, p=0.000					
Household head occupation					
Primary	53.7	16.6	5.1	24.6	21 829
Secondary	36.7	17.8	4.9	40.6	3292
Tertiary	22.7	15.2	5.7	56.5	6166
No occupation	46.0	13.5	4.7	35.9	2844
Pearson χ^2^=2900, p=0.000					
Household head education					
Illiterate	57.0	16.2	5.0	21.7	21 541
Primary	34.3	18.8	6.1	40.9	4331
Secondary	25.4	15.4	5.1	54.1	7194
Higher	11.1	10.8	3.5	74.7	1065
Pearson χ^2^=4200, p=0.000					
Economic status					
Poor	64.0	14.9	4.7	16.5	7154
Non-poor	41.3	16.6	5.3	36.8	7154
Pearson χ^2^=1700, p=0.000					
Caste group					
General	24.7	18.1	5.4	51.9	10 546
OBC	48.7	16.5	5.2	29.7	13 564
SC	61.2	15.0	4.7	19.0	7157
ST	68.2	12.0	5.6	14.3	2864
Pearson χ^2^=3400, p=0.000					
Religion					
Hindu	49.9	16.0	5.1	29.1	27 736
Muslim	29.5	18.0	6.7	45.8	3822
Christian	28.3	16.5	3.2	52.0	2573
Pearson χ^2^=1300, p=0.000					
Region					
North	35.5	17.8	3.9	42.8	7624
Central	64.0	13.3	3.2	19.6	6584
East	53.9	14.9	5.7	25.5	5807
Northeast	4.2	8.4	10.6	76.9	1422
West	38.5	26.0	5.1	30.5	4683
South	39.9	14.0	6.4	39.8	8011
Pearson χ^2^=3500, p=0.000					
Total	46.3	16.2	5.1	32.3	34 131

The transition from the practice of open defecation to the adoption of improved toilet facilities ranges from 11 to 18% across various socio-economic levels between 2004–2005 and 2011–2012. During 2004–2005 and 2011–2012, rural households were more likely than urban households to practice open defecation. More than half (57%) of rural households practiced open defecation, while just (13.7%) of urban households practiced open defecation. The prevalence of improved toilet facilities (65%) was higher among urban households than their rural counterparts (21.6%) during 2004–2005 and 2011–2012. The type of house (*kucha* and *pucca*) may be considered as a proxy for the quality of the house. The sanitation condition by the type of house revealed that nearly 60% of the households residing in *kucha* houses (low quality of floor, walls and roof) and 19% of the households residing in *pucca* houses (better quality of floor, walls and roof) practiced open defecation during 2004–2005 and 2011–2012.

Furthermore, the prevalence of open defecation was remained higher (64%) among poor households than among economically better-off households (41%) during 2004–2005 and 2011–2012. Previous evidence shows that the practice of open defecation better-off households in rural India is considerably higher because the use of latrines is associated with pollution.^20^ Our analysis showed that the prevalence of open defecation is negatively associated with the education level of the head of household. So the practice of open defecation is much higher (57%) among households with an illiterate head of household compared with households with higher-educated household heads (11%). Significant differences were also observed in the practice of open defecation across the caste, religious and occupational groups. For instance, the prevalence of open defecation in 2004–2005 and 2011–2012 was lower among general caste households (25%) compared with OBC (49%), SC (61%) and ST (68%) households. Further, among the religious groups, Hindu households were found to practice open defecation (50%) more as compared with Muslim (30%) and Christian (28%) households. Similarly, households having heads with a primary occupation (54%) had higher open defecation rates as compared with a tertiary occupation (23%).

The practice of open defecation was remained higher in 2004–2005 and 2011–2012 in central (64%) and eastern region households (53%) compared with southern (40%), western (39%), northern (35%), and northeastern households (4%). The transition in the adoption of improved sanitation facilities by households varies significantly across the different socio-economic and regional characteristics of the households (χ^2^ statistics, p<0.001).

### Transition to improved sanitation facilities of households across the major states

Table 2 shows that the prevalence of open defecation was remained higher (70–50%) among households from Orissa, Bihar, Madhya Pradesh, Uttar Pradesh, Rajasthan, Jharkhand, Karnataka and Tamil Nadu in 2004–2005 as well as in 2011–2012. In other states, including Kerala, Delhi, Assam, Jammu and Kashmir, Punjab, Uttaranchal, Gujarat and West Bengal, adoption of improved toilet facilities was higher (93%) in 2004–2005 and 2011–2012. Further, the number of households that adopted improved toilet facilities in 2011–2012 from open defecation in 2004–2005 was higher (45–20%) among the following states: Himachal Pradesh, Maharashtra, Haryana, Chhattisgarh and West Bengal. The number of households that shifted to the practice of open defection in 2011–2012 from improved toilet facilities in 2004–2005 was higher in the states of Assam (15%), Jharkhand (13%), Daman and Diu (13%) and Andhra Pradesh (11%).

**Table 2.** Transition in sanitation behaviour of households from 2004–2005 to 2011–2012 across the major states in India

	Change in households’ sanitation			
Major states	Openly defecated in 2005 and 2012	Openly defecated in 2005 and adopted improved toilet facilities in 2012	Adopted improved toilet facilities in 2005 and defecated openly in 2012	Adopted improved toilet facilities in 2005 and 2012
Jammu and Kashmir	16.8	13.0	1.7	68.5
Himachal Pradesh	22.9	42.0	1.1	34.1
Punjab	14.7	21.6	4.0	59.7
Uttaranchal	28.6	16.0	3.9	51.4
Haryana	25.0	33.0	3.8	38.2
Delhi	8.2	6.1	4.8	80.9
Rajasthan	61.1	11.4	4.6	22.9
Uttar Pradesh	63.8	12.4	3.5	20.3
Bihar	66.2	10.0	5.1	18.7
Assam	5.6	5.8	15.2	73.5
West Bengal	34.3	22.2	3.4	40.2
Jharkhand	60.6	8.1	13.4	18.0
Orissa	70.5	12.5	5.6	11.5
Chhattisgarh	63.0	22.4	0.8	13.9
Madhya Pradesh	64.9	10.4	3.6	21.1
Gujarat	39.0	15.0	6.2	39.8
Daman and Diu	39.1	31.0	12.9	17.1
Maharashtra	39.0	31.4	4.5	25.2
Andhra Pradesh	38.4	15.9	11.4	34.3
Karnataka	54.6	15.6	3.5	26.3
Goa	8.1	16.5	9.8	65.6
Kerala	0.4	4.5	1.9	93.2
Tamil Nadu	50.2	15.3	5.3	29.2
Total (India)	46.9	16.3	5.2	31.7

### Socio-economic determinants of transition to improved sanitation facilities

Table 3 shows the effect of different socio-economic factors on the likelihood of a transition to improved sanitation facilities by fitting the multinomial logistic regression model. For ease of interpretation, we interpreted adjusted the percentages of transition to improved sanitation facilities of the households from multinomial logistic regression and the MCA conversion model by various socio-economic characteristics. Significant differences were observed among the rural–urban and *kucha–pucca* categories of households in the practice of open defecation during 2004–2005 and 2011–2012. Households that continued to practice defecation were more likely to be found in rural areas (53%, p*<*0.01) and in *kucha* houses (55.5%, p*<*0.01) than in urban areas (13.1%) and *pucca* houses (15.6%). Previous evidence has shown that households that have durable and modern houses are more likely to have tap water within the premises and to adopt latrine facilities than households that have low-quality (*kucha*) houses.^22,27^ Similarly, economically poor households were more likely to practice open defecation (61%, p<0.01) than economically well-off households (35%). Economic status is an important determinant in the type of house that is built and in adopting modern sanitation facilities. Economically poor households are more likely to have *kucha* houses and the lack of availability and accessibility of facilities causes them to continue to practice open defecation.^23,25^

**Table 3.** Multinomial logistic regression analysis: adjusted percentage of transition in sanitation behaviour of the households by different socio-economic characteristics

	Change in households’ sanitation			
Background characteristics	Defecated openly in 2005 and 2012	Defecated openly in 2005 and adopted improved toilet facilities in 2012	Adopted improved toilet facilities in 2005 and defecated openly in 2012	Adopted improved toilet facilities in 2005 and 2012^a^
Place of residence				
Rural	53.0***	18.2***	4.6***	24.2
Urban^a^	13.1	14.3	4.5	68.1
Type of house				
*Kucha*	55.5***	18.4***	4.4***	21.7
*Pucca* ^a^	15.6	14.6	5.0	64.9
Household head occupation				
Primary	48.9	17.8***	4.6	28.7
Secondary	31.4	17.6***	5.0	46.0
Tertiary	17.3***	14.7	4.6	63.4
No occupation^a^	39.5	14.9	4.4	41.2
Household head education				
Illiterate	51.2***	18.0***	4.6***	26.2
Primary^a^	31.2	18.0	5.2	45.6
Secondary	19.8	14.5***	4.6	61.1
Higher	8.1***	8.7***	3.3	79.9
Economic status				
Poor	60.6***	15.4***	4.4***	19.6
Non-poor^a^	35.4	17.4	4.7	42.5
Caste				
General^a^	21.1	18.1	4.8	55.9
OBC	43.6***	16.9***	4.6	35.0
SC	54.9***	17.6***	4.6***	22.9
ST	64.0***	11.6***	4.2***	20.2
Religion				
Hindu^a^	44.8	17.2	4.6	33.5
Muslim	23.9***	16.5***	5.7***	53.9
Christian	22.0***	16.0***	3.3***	58.8
Region				
North^a^	30.1	22.0	3.6	44.3
Central	59.9***	14.3	2.9**	22.9
East	48.3***	14.3	5.4***	32.0
Northeast	1.8***	7.5***	7.9**	82.9
West	38.5***	25.5***	5.4***	30.5
South	37.7***	13.1***	5.5***	43.7
Total	40.7	17.0	4.6	37.7
Number of observations=34 131			Log likelihood=−30 496.459	
LR χ^2^ (57)=19 330.99				
Prob > χ^2^=0.0000				
Pseudo R^2^=0.2407				

^a^Reference category.

*p<0.1, **p<0.05, ***p<0.01.

Continued practice of open defecation is negatively associated with the education level of the head of the household as well as with the occupation of the head of the household. For instance, the households led by an illiterate head reported more open defecation (51%, p<0.01) than households with higher education levels (8%, p<0.01). Likewise, households with the head’s occupation in tertiary activity reported less practice of open defecation (17%, p<0.01) compared with a primary occupation (49%). Practice of open defecation varies among the social and religious groups. ST (64%, p<0.01), SC (55%, p<0.01) and OBC (44%, p<0.01) households were more likely to practice open defecation as compared with general caste households (21%). Similarly, Hindu households were more likely to practice open defecation (45%) than non-Hindu households (Muslim 24%, p<0.01; Christian 22%, p<0.01). In rural India, Hindu households feel that emptying the pit latrine is associated with the work of lower castes. Higher castes avoid emptying pit latrines because of the associated social stigma, while lower castes resist emptying pit latrines for equal rights, which makes them adopt open defecation.^20^ In the regional categories of households in India, central (60%, p<0.01) and eastern (48.3%, p<0.01) households are more likely to practice open defecation than northern households (30%). The socio-economic status of households in these regions is lower compared with the national average and other regions in India, which is a barrier to adopting latrine facilities.^30^

Results indicated that households that have switched to improved toilet facilities in 2011–2012 from the practice of open defecation in 2004–2005 are more likely to be rural (18%, p<0.01), live in a *kucha* house (18%, p<0.01), illiterate (18%, p<0.01) and with a primary occupation (18%, p<0.01) than their counterparts who are urban (14%), live in a *pucca* house (14%), have a higher education (8.7%) and no occupation (15%). This is mainly because there is a greater proportion of households from rural areas, in *kucha* houses, illiterate and in primary occupations under poor sanitation conditions (open defecation) in the base year (2004–2005). Therefore their share is greater among those who have moved from the practice of open defecation to the adoption of improved toilet facilities. Another reason might be that more people benefit from sanitation policy intervention by the government in lower socio-economic groups than in other groups. Switching to improved toilet facilities in 2011–2012 from the practice of open defecation in 2004–2005 is less likely to be lower for ST, SC and OBC households (11.6%, p<0.01), as well as economically poor households (15.4%, p<0.01) compared with general caste (18%) and non-poor households (17.4%). The movement from the practice of open defecation to the adoption of improved toilet facilities is less among ST households even though their share is greater under poor sanitation (practice of open defecation) in the base year (2004–2005) because they were socially excluded from getting benefits from government policies. An economically poor household's share is lower under poor sanitation conditions (practice of open defecation) in the base year (2004–2005) than non-poor households, therefore their movement is less from the practice of open defecation to the use of improved toilet facilities. According to regional categories of households in India, the transition to improved toilet facilities in 2011–2012 from the practice of open defecation in 2004–2005 is more likely to be from the western region (25%, p<0.01) compared with the northern region (22%).

Movement of households from improved toilet facilities to the practice of open defecation is more likely among rural (4.6%, p<0.01), illiterate (4.6%, p<0.01) and eastern region (5.4%, p<0.01) households compared with urban (4.5%), higher educated (3.3%) and northern region households (3.6%).

### Effect of transition to improved sanitation facilities on diarrhoeal morbidity

**Figure 1:**
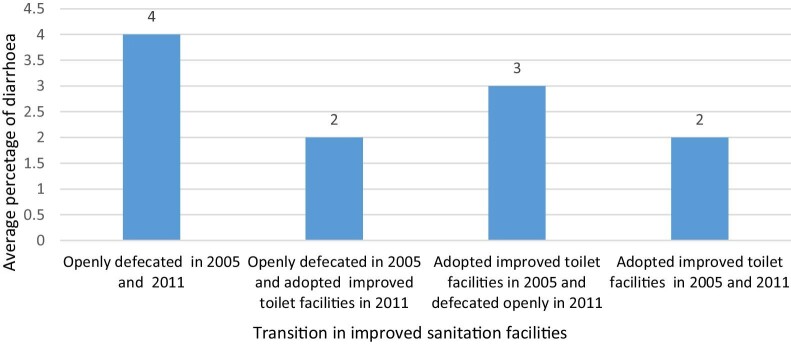
The average percentage of household members who fell ill due to diarrhoeal morbidity by change in the household’s sanitation in India.

Figure 1 shows the average percentage of household members who fell ill due to diarrhoeal morbidity by the change in households’ improved sanitation facilities in India. The average percentage of household members who fell ill due to diarrhoeal morbidity was higher (4%) among the households that practiced open defecation continuously in both periods (2004–2005 and 2011–2012) compared with the households who adopted improved toilet facilities during the same periods (2%). The average percentage of household members who fell ill due to diarrhoeal morbidity declined (2%) among the households that adopted improved toilet facilities in the recent period (2011–2012) from the practice of open defecation in 2004–2005. The average percentage of household members who fell ill due to diarrhoeal morbidity has increased (3%) among the households that switched to open defecation in 2011–2012 from improved toilet facilities in 2004–2005. It is clear that the average percentage of household members who fell ill due to diarrhoeal morbidity was low and it has been reduced with the continued adoption of improved sanitation facilities.

**Figure 2:**
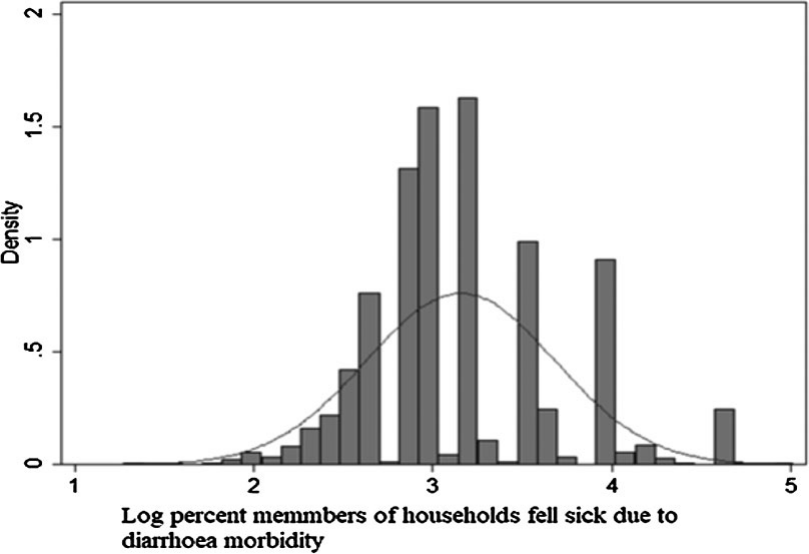
Normal probability curve for percentage of household members who fell ill due to diarrhoeal morbidity.

Figure 2 shows the normal probability curve for the percentage of household members who fell ill due to diarrhoeal morbidity is normally distributed. Therefore we performed a multivariate linear regression for our analysis. Table 4 presents the multivariate linear regression effect of the transition to improved sanitation facilities on the average percentage of household members who fell ill due to diarrhoeal morbidity, net other covariates. The results show that the average percentage of household members who fell ill due to diarrhoeal morbidity has declined significantly (β=−0.06, p<0.04) among the households that have switched from the practice of open defecation in 2004–2005 to improved toilet facilities in 2011–2012. Diarrhoeal morbidity was significantly lower (β=−0.09, p<0.001) among the households that adopted improved toilet facilities in both time periods (2004–2005 and 2011–2012) compared with households who practiced open defecation during the same period. In terms of religious status of the households, the percentage of Muslim household members who fell ill due to diarrheal morbidity was significantly lower (β=−0.14, p<0.001) compared with Hindu households.

**Table 4.** Multivariate linear regression of percentage of sick members of household due to diarrhoeal morbidity by transition in sanitation behaviour of the households and socio-economic variable

Background variables	β coefficient	p-Value	95% CI of β	
Transition in sanitation				
Defecated openly in 2005 and 2012^a^				
Defecated openly in 2005 and adopted improved toilet facilities in 2012	−0.06	0.03	−0.12 to 0.00	
Adopted improved toilet facilities in 2005 and defecated openly in 2012	0.01	0.90	−0.09 to 0.10	
Adopted improved toilet facilities in 2005 and 2012	−0.09	0.00	−0.15 to 0.03	
Place of residence				
Rural^a^				
Urban	0.03	0.23	−0.02 to 0.09	
Type of house				
*Kucha*^a^				
*Pucca*	−0.01	0.66	−0.06 to 0.04	
Occupation				
No occupation^a^				
Primary	0.01	0.78	−0.06 to 0.09	
Secondary	0.03	0.54	−0.07 to 0.12	
Tertiary	0.04	0.38	−0.05 to 0.13	
Education				
Illiterate^a^				
Primary	0.02	0.62	−0.05 to 0.08	
Secondary	−0.02	0.45	−0.08 to 0.04	
Higher	0.11	0.16	−0.04 to 0.26	
Economic status				
Poor^a^				
Non−poor	0.08	0.001	0.04 to 0.13	
Caste				
General^a^				
OBC	−0.03	0.32	−0.08 to 0.03	
SC	−0.02	0.42	−0.09 to 0.04	
ST	−0.03	0.53	−0.12 to 0.06	
Religion				
Hindu^a^				
Muslim	−0.14	0.001	0.20 to 0.08	
Christian	0.09	0.09	−0.01 to 0.18	
Region				
North^a^				
Central	0.04	0.16	−0.02 to 0.10	
East	0.05	0.17	−0.02 to 0.13	
Northeast	0.13	0.07	−0.01 to 0.27	
West	0.06	0.12	−0.01 to 0.13	
South	0.18	0.001	0.12 to 0.25	
Constant	3.09	0.001	2.99 to 3.18	
Total number of observations	2701			
R^2^	0.0336			
Adjusted R^2^	0.0256			
Prob > F=	0.00 001			

^a^Reference category.

CI: confidence interval.

## Discussion

Utilization of modern sanitation facilities is a significant predictor of diarrhoeal morbidity. Our findings reveal that the average percentage of household members who fell ill due to diarrheal morbidity was significantly lower among the households that continued to adopt improved toilet facilities in 2004–2005 and 2011–2012 compared with the households that continued the practice of open defecation during the same period, after controlling for socio-economic covariates. An increase in the availability of modern sanitation facilities among households in 2011–2012 compared with poor sanitation in 2004–2005 resulted in a significant decrease in diarrhoeal morbidity. Our findings are consistent with previous cross-sectional studies and longitudinal studies suggesting that improved sanitation reduces diarrhoeal morbidity.^6–9^,^27,28,31^ However, the cross-sectional studies cannot assess the transition in sanitation conditions of the same households and their effect on diarrhoeal morbidity over a period of time. As a result, the benefits of our findings over cross-sectional studies are that the dynamics of changes in household sanitation facilities from open defecation to adoption of improved sanitation facilities reduce diarrhoeal morbidity.^28^ In contrast to our results, interventional evidence showed that a reduction in open defecation did not show any significant impact on diarrhoea among children, due to the fact that the threshold level of open defecation was not reached in the interventional area.^34^ Our findings suggest the proportion of households who adopted improved sanitation facilities over a period of time was considerable, as seen by the reduction of diarrhoeal morbidity among household members compared with households that practiced open defecation.

During the period 2004–2012, the overall transition from the practice of open defecation to the adoption of improved toilet facilities was marginal across different socio-economic characteristics of households. However, improvement in the accessibility of household sanitation facilities over that period of time was not consistent with an improvement in the standard of living. Previous studies have shown that there was a substantial decrease in the poverty rate (17%) during that same period.^32^ Sanitation behaviour of households in India goes beyond the development indicators. Most often, household sanitation behaviour is associated with ritual purity, pollution and caste among Hindu households.^17,18^ However, the transition to modern sanitation facilities from the earlier practice of open defecation was greater in rural areas, in *kucha* houses, the illiterate and those in a primary occupation compared with their counterparts in urban areas (*pucca* houses, highly educated and tertiary occupations). Greater progress in rural areas with household characteristics such as illiteracy and primary occupations indicates that they had more room for improvement than their better-off counterparts. This is primarily because these households had poorer sanitation conditions in the base period of 2004–2005. In addition, affirmative action programs might have contributed to greater progress in disadvantaged groups. During 2004–2012, 64.3 million individual household latrines were constructed for below-poverty-line households as part of the government of India's flagship program ‘Total Sanitation Campaign’, launched in 1999. The aim of this program was to accelerate sanitation coverage and to make rural India ‘open defecation free’ by the end of 2017. This program might have played a critical role in the adoption of improved toilet facilities in socio-economically disadvantaged households.^26^ Nevertheless, the movement from the practice of open defecation to the adoption of improved toilet facilities is less among ST households, even though their share is greater under poor sanitation (practice of open defecation) in the base year (2004–2005). This may be attributed to the geographical location of ST households, which may lead to social exclusion in receiving benefits from government policies.^18^

Despite progress from open defecation to the adoption of improved toilet facilities across socio-economic groups, after controlling other covariates, our results show that households in rural areas with housing characteristics like *kucha* houses and illiterate heads of households, which are highly concentrated in the central and eastern regions of the country, continued to show greater open defection than their counterparts. Previous evidence shows that in states such as Madhya Pradesh, Uttarpradesh and Bihar in the central and eastern regions, households prefer open defecation because it is a pleasure, comfortable and convenient, rather than the use of toilet facilities.^33^ In rural India, access to *pucca* houses and basic sanitation facilities continued to be low among SC, ST and illiterate households, as they have low social capital and are socially excluded from society, creating a barrier to the adoption of improved toilet facilities. Previous literature has shown that they do not have sufficient income to cover the cost of latrine construction, despite of the government's target to provide subsidised latrine facilities.^18^ In addition to that, social stigma in the Hindu religion is associated with emptying pit latrines in rural areas, and the lower castes resist emptying of pit latrines for equal rights, which makes them practice open defecation.^17,19,20^ This evidence supports findings from multivariate linear regression that Hindu households have significantly higher morbidity due to poor sanitation than Muslim households after controlling for other factors. Another possible reason is some of these households, like rural, illiterate and in eastern regions, are switching from improved toilet facilities to open defecation because of the poor quality of latrine facilities that were provided by the government. They do not have durable modern houses or accessibility to running water within the premises. Another reason is the availability of improved sources of drinking water and sanitation has improved over time, but the accessibility of these services has not improved over time among these groups and regions.^25^

Despite the fact that some of the households moved from the practice of open defecation to adoption of improved sanitation facilities marginally across socio-economic groups, the movement of some households from improved toilet facilities to open defecation is more likely among rural, illiterate and eastern region households compared with urban, higher-educated and northern region households. It clearly indicates that even though they have access to toilet facilities, they still practice open defecation. Our findings, like previous studies, show that people practice open defecation even when they have access to toilet facilities. This is due to the poor quality of toilet facilities provided by the government, as well as the fact that people prefer open defecation, especially in rural areas, because it is convenient, pleasant and comfortable. Another reason is that a large amount of water is needed to properly maintain latrines.^33^

## Conclusions

Our results have a few obvious limitations, such as caveats associated with self-reported morbidities and a lack of information about toilet usage, preference and other behavioural factors that determine the use of toilet facilities. Our study is confined to longitudinal analysis of transitions in household sanitation facilities and their effect on diarrhoeal morbidity; it is not an interventional study. Therefore we cannot be certain of the impact of change in improved sanitation facilities on morbidity. Other factors that may affect diarrhoeal morbidity, such as safe disposal of child faeces and mothers’ hand washing behaviour were not considered in this study. Our analysis is confined to the household level, which means the average percentage of all household members affected by diarrhoeal morbidity with respect to changes in household sanitation facilities. We did not consider diarrhoeal morbidity in children at the household level, due to small samples and a large number of missing cases.

This study significantly contributes to policy and planning of water, sanitation and hygiene (WASH) programs in India. Regarding the findings of this study, we suggest providing inclusive sanitation for all. The ongoing Cleans India mission should focus on lower socio-economic groups in rural areas and the central and eastern regions of India to improve sanitation facilities and reduce the associated disease of diarrhoea. Furthermore, the construction of *pucca* houses and improving the source of running water within the premises are key for the adoption of improved sanitation, whereas purity, pollution and other cultural factors are barriers to the adoption of improved sanitation. Therefore policies should focus on these factors to adopt modern sanitation facilities in India.

## Data Availability

The data underlying this article will be shared on reasonable request to the corresponding author. However, IHDS wave-1 and wave-2 panel data is available in the public domain.
